# Real-time imaging through strongly scattering media: seeing through turbid media, instantly

**DOI:** 10.1038/srep25033

**Published:** 2016-04-26

**Authors:** Sriram Sudarsanam, James Mathew, Swapnesh Panigrahi, Julien Fade, Mehdi Alouini, Hema Ramachandran

**Affiliations:** 1 Raman Research Institute, Sadashiv Nagar, Bangalore, 560080, India; 2 Institut de Physique de Rennes, Universite de Rennes 1 CNRS, Campus de Beaulieu, 35042 Rennes, France

## Abstract

Numerous everyday situations like navigation, medical imaging and rescue operations require viewing through optically inhomogeneous media. This is a challenging task as photons propagate predominantly diffusively (rather than ballistically) due to random multiple scattering off the inhomogenieties. *Real-time* imaging with ballistic light under continuous-wave illumination is even more challenging due to the extremely weak signal, necessitating voluminous data-processing. Here we report imaging through strongly scattering media in real-time and at rates several times the critical flicker frequency of the eye, so that motion is perceived as continuous. Two factors contributed to the speedup of *more than three orders of magnitude* over conventional techniques - the use of a simplified algorithm enabling processing of data on the fly, and the utilisation of task and data parallelization capabilities of typical desktop computers. The extreme simplicity of the technique, and its implementation with present day low-cost technology promises its utility in a variety of devices in maritime, aerospace, rail and road transport, in medical imaging and defence. It is of equal interest to the common man and adventure sportsperson like hikers, divers, mountaineers, who frequently encounter situations requiring realtime imaging through obscuring media. As a specific example, navigation under poor visibility is examined.

The difficulty in viewing the shore from a boat at sea on a foggy day, and the inability to directly see a bullet lodged in flesh are examples where recurrent random scattering in an inhomogenous medium degrades the image-bearing capability of light. The normally ballistic transport of photons is rendered diffusive, precluding direct viewing of the source and inhibiting formation of images (or shadows) of intervening objects. The need for imaging through scattering media, and to do so in real-time, continues to spawn fresh research even today^1^,^2^,^3^,^4^, as it has proven difficult to obtain a technique that is simple, fast, compact and portable while also being versatile and inexpensive.

The various approaches pursued over the years^5^,^6^,^7^ either extract the minute amount of ballistic (forward scattered) photons from the overwhelmingly large amount of diffusive photons, or examine the diffusive photons themselves. Intuitively obvious is the technique of time-gated ballistic imaging where an ultrashort pulse of light (~100 fs) illuminates the sample and the varying transit times of photons enables selection of ballistic light. However, a far simpler and inexpensive approach is one where a continuous-wave source is modulated in intensity or polarisation and ballistic photons in the emergent light are identified based on their retention of the periodic modulation as opposed to the uniform temporal strength of the diffusive photons and ambient light. The exceedingly small proportion of ballistic photons necessitates the acquisition of emergent light over a certain length of time, and a Fourier transform of the time-series enables their extraction to form two-dimensional images, as was first demonstrated in ref. 8. This method of source modulation and Fourier-transform-based discrimination has now been extended to numerous modalities^9^,^10^,^11^,^12^,^13^,^14^,^15^,^16^. However, despite availability of well-optimized codes for Fourier analysis, real-time imaging, even for moderate scattering, remains elusive as the large number of pixels in an image and the length of the time-series required to be examined render the computation voluminous.

A solution to this long-standing problem is provided here by use of a simplified algorithm - the quadrature lock-in discrimination (QLD) - for ballistic light extraction, coupled with efficient data-routing and hybrid parallelisation of tasks, that enable simultaneous data acquisition and processing, leading to real-time display of images with low latency and at rates faster than the eye can perceive. The utility and versatility of the technique is demonstrated in table-top experiments that simulate scenarios commonly encountered in navigation. A scene containing several light sources is viewed through a scattering medium simulating the view from an aircraft approaching a city airport on a foggy day. The utility of modulated light sources and the efficacy of QLD in the elimination of clutter for the unambiguous identification of runway lights in the presence of other distracting sources is demonstrated, on the one hand, and in the easy and rapid visualisation of the runway under poor visibility on the other. Thereafter, a passive scene is illuminated by modulated light, and the technique used to see both stationary and moving objects hidden from view, affirming practical utility of the technique in real-life situations.

## Quadrature Lock-in Discrimination

When the frequency of modulation of the source is unknown, a fast Fourier transform (FFT) on the time series provides the amplitudes at various frequencies enabling the selection of the dominant one. *A priori* knowledge of the modulation frequency, however, eliminates the need for spectral decomposition - a fact utilised by electronic lock-in detectors to extract weak signals from noise. Time integration of the product of the recorded time-series with an appropriately phase shifted reference sinusoid yields an output proportional to the strength of the component at the reference frequency present in the original data. However, obtaining a two-dimensional image using a photo-detector and electronic lock-in detector requires a step-scan over the array of pixels, as in ref. 17. The same idea may be implemented in software over an entire image, but the computational complexity remains as the phase difference between the modulating and the reference sinusoids has to be determined. Multiplying two sinusoids of the same frequency produces an output proportional to the cosine of the phase difference between them, and thus phase matching is desirable. The technique of Quadrature Lock-in Detection (QLD) circumvents the problem of the actual phase determination very simply, by making a copy of the time series, and multiplying one by the reference sinusoid, and the other by a sinusoid 90° phase shifted to this, so as to obtain the in-phase and quadrature-shifted components at the frequency of interest, both of which are then used to determine the amplitude at the frequency of interest^18^.

**Figure 1:**
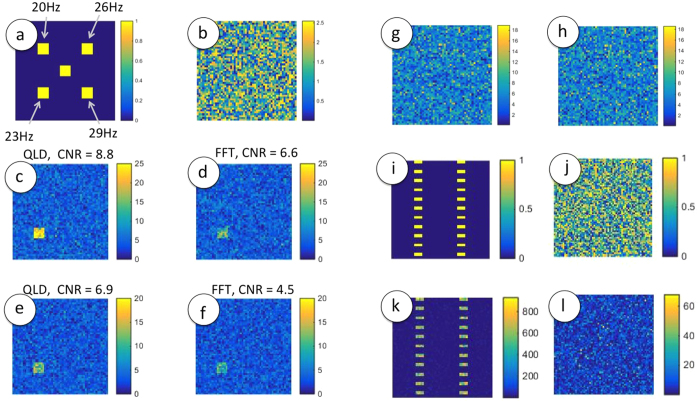
Imaging through scattering medium by use of QLD and FFT techniques in a numerical example, and a suggested use of QLD for navigation. (**a**) A frame containing five sources of light (intensities of four of which are modulated sinusoidally while one is held fixed), depicted at an instant when all sources are at their maximum intensity. In this example, the frequencies of modulation are 20, 23, 26 and 29 Hz, and the frequency of interest is 23 Hz. (**b**) A representative “raw data” frame where the random noise has been added to each pixel in the frames of the type (**a**), simulating a frame recorded on the camera when the sources are viewed through fog. (**c**) Processed image, obtained using QLD, of the time series formed from N (82) frames of the type (**b**), displays a superior contrast-to-noise ratio in comparison to (**d**) which is obtained using FFT on the same N frames as in (**c**). (**e**) Processed image with the source of interest clearly seen, obtained by performing QLD on N = 41 frames of the type (**b**). (**f**) Processed image obtained by FFT, using the same N = 41 frames as in (**e**). (**g**,**h**) Images obtained using QLD and FFT techniques, at an “incorrect” frequency, 31 Hz, for the same data used in (**c**,**d**). QLD can be used, as shown in this computer simulated example, to detect modulated runway lights (**i**), which cannot be directly viewed under foggy situation (**j**). QLD at the correct frequency enables picking out the lights (**k**), which do not show up when QLD is performed at a different frequency (**l**). Details of simulation are given in Supplementary Information.

We first compare the performance of QLD and FFT through numerical simulation of intensity-modulated light sources being viewed through fog. In the absence of a scattering medium, the imaging camera would acquire, over the distinct regions corresponding to the images of the light sources, intensities varying sinusoidally at the respective frequencies shown for a particular instant in Fig. 1a. In the presence of a scattering medium, the photons follow diffusive trajectories and are grossly deviated from their original paths; ballistic photons are significantly reduced in number. Every pixel in the camera now receives diffusive light. The regions corresponding to the direct images of the sources too, receive predominantly diffusive light, along with a very small amount of ballistic light, the intensity of which decreases exponentially with the strength of scattering. Consequently, each recorded frame shows diffuse illumination, with no discernible feature as in Fig. 1b. A sequence of such “raw data” frames were generated numerically and QLD and FFT were performed on them (details in Methods). While the five sources are hidden from direct view in any typical raw data frame, the appropriate source is revealed when QLD (or FFT) examines one of the modulating frequencies, as depicted in Fig. 1c–f. However, when the examined frequency does not match any of the modulating frequencies, no source is visible, e.g., Fig. 1g,h. It is seen that QLD offers a better noise rejection, yielding a higher contrast-to-noise ratio (CNR, see Supplementary Information) in comparison to FFT, for the same input data. For example, QLD on a time- series of 82 frames yields a CNR of 8.8, while FFT 6.6. In fact, QLD on N/2 frames yields a CNR comparable to FFT on N frames.

This simple yet effective technique may be put to use, for example, in navigation. Let us consider an airfield where the runway lights are modulated at frequency *ω*_*o*_, that in the presence of fog, become obscured from view, as depicted in Fig. 1j. A time series of such raw-data frames is acquired and QLD is performed over them to extract the ballistic information. The runway lights are reconstructed (with a slight noise) when QLD is performed at the correct frequency; these lights do not show up in the processed image when QLD is performed at the incorrect frequency, as is evident from Fig. 1k, 1l.

A major advantage of such standard QLD approach is its immunity to the phase difference between the modulation at the source and the observer^19^,^20^. The need for frequent phase determination that becomes necessary when the modulation has phase jumps, or in the presence of relative motion between source and observer, is now eliminated, making lock-in detection less cumbersome and more reliable. As phase search is no longer required, QLD leads to a drastic reduction in computation, and hence faster image retrieval. The superior noise rejection of QLD, which directly translates to use of shorter time series, further contributes to reduction in acquisition and processing time. In addition, it doubles the bandwidth for the rate of modification of a scene (change in relative phase/motion between source and observer). Another feature of great relevance is that the entire time-series has to be acquired for FFT to be performed. In contrast, processing may begin with the acquisition of the first frame, in the case of QLD - a fact that contributes significantly to the reduction of latencies. All these aspects are extremely important in applications where speed is of essence, as in navigation. Practical utility demands that images be produced with near zero latency, that is, with negligible delay between the acquisition of data and the rendering of the image. Images must also be produced at rates faster than 30 frames per second (fps) to mimic natural vision with continuity of motion, and at twice that rate to avoid flicker (perception of variation in intensity)^21^. Here we report implementation of QLD in software, performed at the modulation frequency, which provides the advantage of versatility, low cost and upgradability, while providing processed images in real-time with latencies of the order of milliseconds, and frame rates of 100 fps, limited only by the performance of the camera.

## Processing Time and Speedup

**Figure 2:**
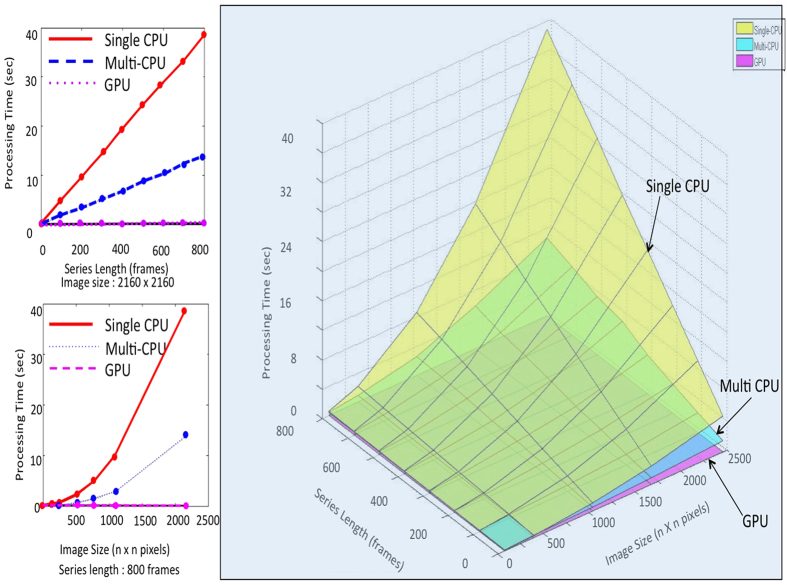
Latencies for QLD when computed using a single CPU (yellow), multi-CPU of 4 cores with hyperthreading, resulting in a total of 8 threads (blue) and a single GPU (pink).

As the Fourier decomposition is essentially a series of QLDs being performed at different frequencies, it is expected that QLD at a *single* frequency can be executed faster. However, several Fast Fourier Transform (FFT) algorithms have been developed that compute the Fourier transform not by the definition, but by methods of reducing the problem to smaller parts, so that the computation time is reduced from *O*(*N*^ 2^) to *O*(*NlnN*), N being the length of the time series. Heavily optimised commercial codes are available that may be used for minimising processing time, and thus, it may well be that a FFT consumes lesser time than QLD (though at the cost of efficiency of spectral decomposition, as evidenced by the lower CNR for FFT in Fig. 1). We examined, as function of the size of the frame and the length of the time- series, the time taken to obtain a processed image using FFT and QLD algorithms in MATLAB (see Supplementary Information). The time advantage of QLD over FFT was minor, implying that the FFT algorithm is indeed well optimised. Next, a comparison was made of implementation of QLD using MATLAB and C++; a speedup of nearly an order of magnitude was observed in the latter. The processing time, nevertheless was still a few seconds. Clearly, such long processing times, with additional overheads of other tasks, cannot be tolerated if real-time images are sought. The phrase “real-time” allows for latencies of different durations in different contexts. For example, while imaging a static scene, a time gap of ~10 *s* may be tolerated. In medical applications, a latency of a few seconds qualifies as real-time. In fact, most often, “real-time” is taken to imply that acquisition is not halted to enable processing and data is being continuously updated, albiet with a latency of several seconds (e.g. ref. 22). Navigation, however, imposes much more stringent demands on the speed of processing. A boat approaching the coast, or a train moving at slow speed, would have moved several 10’s of meters in a few seconds. Thus, a delay of the order of ~ seconds in obtaining an image cannot be afforded; image latencies have to be reduced by at least two orders of magnitude. Further, as smooth movements require a display rate of 25 fps or more, and flicker-free viewing 60 fps or more, it is imperative that the display of processed images and therefore the recording of raw data be carried out at these rates or faster and that the processing be completed within the time interval between acquisition of two frames, which is ~15 milliseconds. In the work presented here, this demand is met by parallelisation of tasks. Present-day desktop computers boast of multicore Central Processing Units (CPUs) and efficient Graphics Processing Units (GPUs), both of which allow for parallelization, but by different means. Multicore CPUs enable simultaneous execution of different tasks running in different threads, working either individually, or in groups, and thus are efficient for *task* parallelization. On the other hand, GPUs, with their very large number of cores, facilitate simultaneous execution of the same operation on an array of data - the so-called Single Instruction Multiple Data (SIMD) processing, offering *data* parallelisation. The time taken for performing QLD on a given set of data using a single CPU, multi-CPU and a GPU are shown in Fig. 2. The use of 4-CPUs with a total of 8 threads has reduced the processing time from 38.7 s to 14 s for 800 frames of size 2160 × 2160. The use of a single GPU for computation brought the computation time down to a mere 20 ms – a gain in speed by a factor of 700 over multi-CPU implementation and of *more than three orders of magnitude* over the single-CPU implementation.

Thus far, only the computational time with the respective algorithms has been considered. However, the task of obtaining a single processed image consists of the acquisition of requisite number of raw frames, transfer of data from the camera to the computer, the performing of QLD by software, and the display and storage of the processed image. In fact, it turns out that with GPU implementation of QLD, computation consumes a very small fraction of the entire time. It now becomes imperative that the time taken by the different non-computational tasks be considerably reduced. In this context, a very important fact is that the C++ environment permits direct access to the camera control (ANDOR Neo sCMOS camera, see Supplementary Information) facilitating rapid setting of camera’s operating parameters, data routing, memory management and display on the computer screen, all of which contribute to time overheads, though to varying extents, and have to be carefully optimised. Leveraging the distinct advantages of CPUs and GPU; we employ hybrid parallelisation where multiple CPU cores are employed for different tasks such as acquisition, buffer management and kernel calls and the GPU is used to perform QLD on the arrays of pixel data, invoking SIMD (see Supplementary Information). With this strategy, we have been able to produce processed images of objects originally obscured by scattering in real-time, in a sustained manner. Frames of size 600 × 600 pixels (3.6 mega-pixels) are displayed at 100 fps, with the first processed image appearing 55 ms after the acquisition of the first raw data frame, and 5 ms after the acquisition of the last raw data frame of the sequence of images required for QLD. This near-zero latency and camera-limited frame rate, that can be maintained over long term operation, paves the way for real-time imaging in scenarios where speed is of essence, as in navigation.

## Experimental Demonstration

**Figure 3:**
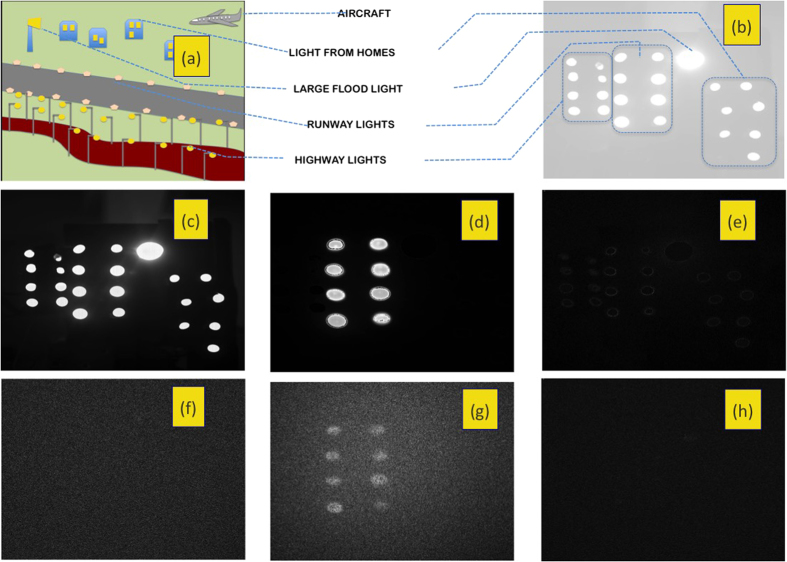
Real-time imaging of light sources obscured by scattering medium: (**a**,**b**) The scene being simulated experimentally is one that has a variety of light sources. (**c**) The scene as it would appear to a pilot approaching for landing on a clear night. (**d**) Image obtained using QLD, where only the modulated runway lights are picked up. (**e**) No light source shows up when QLD is attempted at the incorrect frequency. (**f**) Typical view on a foggy night. (**g**) QLD at the correct frequency shows the runway lights. (**h**) QLD at the incorrect frequency shows no source.

We now demonstrate such real-time imaging in several scenarios, in tabletop experiments that mimic every-day situations. Uncollimated, incoherent light emitting diodes (LEDs), emitting in the orange-red region of the spectrum (600–700 nm) were used as the sources of light. These were powered by dc sources, and the desired LEDs could be modulated in intensity at the required frequency. The use of QLD essentially requires a sinusoidal modulation of the source in some form, e.g., intensity modulation. However, other considerations may require that the intensity remain uniform. In such cases, one may modulate the direction of linear polarisation of the source in a sinusoidal fashion and perform a polarisation based QLD on the emergent light after passing it through a fixed analyser^8^. Discrimination is based on the fact that the ballistic, or the forward scattered light retains its polarisation, while the diffusive light and ambient light are depolarised to various extents. The experiments discussed below, however, employed intensity modulation. The scene was viewed using the ANDOR Neo sCMOS camera and the data acquisition and real-time processing was performed using the parallel processing procedure described in Supplementary Information. Frames of size 600 × 600 pixels, with exposure time 5 ms were recorded at 100 fps. Shorter exposures could be used when the scattering was less. The first scenario mimics the pilot’s view while approaching a runway for landing. In addition to the runway lights, there is a clutter of other sources of light - streetlights, lights in buildings, vehicular lights, etc. as illustrated in Fig. 3a–c. Modulation of the runway lights, either in intensity or in polarisation, enables the use of QLD to reject all light except that from the sources of interest. These alone appear in the processed image when QLD is attempted at the modulation frequency, and are not visible at any other frequency (see Figs. 3d, 3e). Thus, QLD may be used, even when visibility is good, to identify particular sources of interest in the presence of a large number of unwanted sources in the field of view. Next, we simulate a foggy day (or night) where the same set of sources (runway lights and other sources) is obscured as in Fig. 3f, that is, the lights cannot be discerned in snapshots of the scene. This was achieved by interspersing between the scene and the camera a glass container with a scattering medium, that has spherical polydisperse scatterers, ranging in size from 0.5 *μ*m to 5 *μ*m, typical of water droplets in atmospheric fog. The scattering medium simulated 262 m of moderate fog, or 26 m of dense fog (see Supplementary information). QLD at the correct frequency reveals the light sources of interest as may be seen in Fig. 3g. No light source shows up when QLD is performed at a different frequency, e.g., Fig. 3h. Processed images of size 600 × 600 pixels were obtained at 100 fps, with a latency of 5 milliseconds. Thus, with regard to continuity of motion and immediacy of view, the processed images appear to the pilot akin to normal vision.

**Figure 4:**
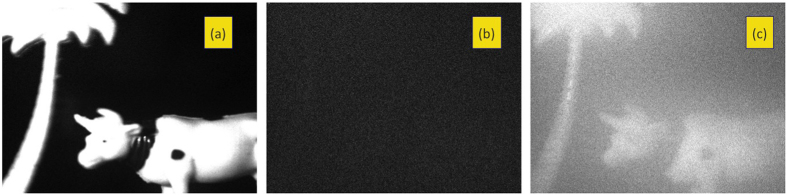
Real-time imaging through scattering medium: (**a**) Model of a tree and a cow, kept in the field of view. (**b**) A typical camera image when the tree and the cow are obscured by a strong scattering medium interspersed between the scene and the observer. (**c**) Upon QLD, the tree and the cow become discernible.

The second scenario simulated was one often encountered while driving in the countryside in fog. Unknown terrain, a curve in the road, a tree in the path, a boulder fallen onto the road, cyclists, or even animals in the path cannot be made out from the raw images of the scene captured by a camera on the vehicle. If, however, the illumination (car fog-lights, say) is modulated in intensity or polarisation, and realtime QLD performed on the captured images, these objects can easily be made out, as illustrated in Fig. 4. Models of a tree and an animal, shown in Fig. 4a, were placed in the field of view of the camera. Fog, simulated by the strongly scattering medium described earlier, obscured these from view as is evident from Fig. 4b. However, performance of QLD enables one to see these objects as in Fig. 4c. These images were also obtained in realtime at 100 fps, on processing data acquired over 50 milliseconds. Sharper contrast can be attained by processing over a longer time-series. The same display frame-rate of 100 fps can be maintained; the first processed image would appear after a longer delay from the first acquired raw data frame, though the gap between the last acquired raw data frame and appearance of the first processed image would still be ~5 ms.

**Figure 5:**
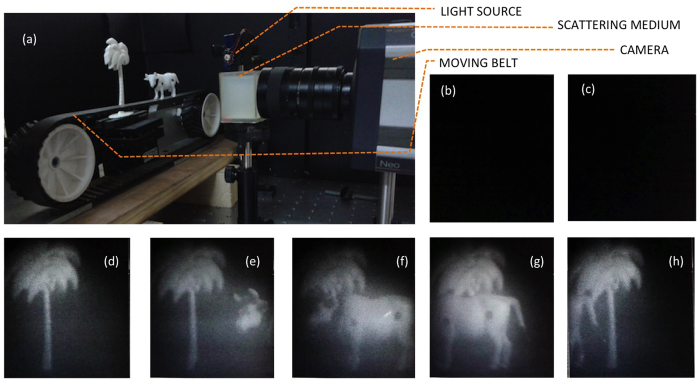
Realtime QLD imaging of a moving object. (**a**) The experimental setup where models of a tree and a cow illuminated by a modulated source, though in the field of view of a camera, are obscured due to an intervening scattering medium. The cow is on a moving belt so that it moves across the scene from right to left. (**b**) A typical raw data frame recorded by camera, (**c**) processed QLD image when performed at the “incorrect” frequency, (**d**–**h**) snapshots from a real-time movie displaying processed QLD images (at the “correct” frequency), showing the moving object clearly. Processed images of 600 × 600 pixels, are obtained at 100 fps, are obtained 55 ms after recording the first frame and 5 ms after recording the last frame of the time series.

Finally, we demonstrate real-time imaging through scattering media using QLD, when there is a relative movement between the object and the viewer, as would be the case in a navigation. The changing separation between the object and the observer adds to the complexity in modulation-based imaging as this continuously alters the relative phase between source and observer. Thus, to obtained good images, with minimal smearing, it is important that inter-frame delays be low and that images be extracted from shorter time series. The second experiment was repeated, this time with the model of the animal placed on a moving belt; the experimental setup is shown in Fig. 5a. Once again, the scene illuminated by modulated light was viewed through a scattering medium. Figure 5b,c show a typical raw data frame and a processed image when QLD is performed at an “incorrect” frequency. In both cases, no object can be discerned. Figure 5d–g are snap-shots from a movie taken of a computer screen that displays the processed images of QLD performed at the correct frequency. Both the stationary object (tree) and the moving object (cow) can be seen quite well. In this experiment, raw frames were acquired at 100 fps, and processed images generated at the same rate. The latency was 5 ms, hence compatible with realtime flicker-free display to the human eye.

The three experiments show that the technique is capable of providing real-time, clutter-free images through scattering media. While many modern airports have sophisticated instrument landing systems, the technique presented in this article is likely to find utility for various other forms of navigation - small aircraft in private fields, rail and road travel and maritime travel. The technique is equally well applicable to other areas, like imaging through flesh, rescue operations in fires, and deep ocean viewing, that demand the mitigation of effects of multiple scattering and where speed is of essence. Some situations of medical imaging, e.g., looking at a beating heart, impose an upper limit on the time afforded for data capture, while viewing moving objects like a victim in a smoke-filled room^23^, or saving a drowning person, also restricts the time afforded for processing.

To summarise, we have used modulated, continuous-wave incoherent light sources and performed QLD on the light emerging from a strongly scattering medium to discriminate the ballistic light from the diffusive. This, in conjunction with hybrid parallelisation, has enabled, in a sustained manner, the visualisation of obscured sources and hidden objects in real-time, with millisecond latencies and at frame rates far exceeding the usual refresh rate of TV movies (25 fps) and the critical flicker frequency of the human eye (60 fps). The versatility of the technique has been demonstrated in three different scenarios commonly encountered in navigation. By virtue of its simplicity, extremely low cost, and portability, the technique demonstrated here has enormous potential for application, providing an interesting alternative to the well-established time-gated ballistic imaging with pulsed light.

## Methods

### Numerical simulations

The scene being simulated is one that has five light sources, four of which are sinusoidally modulated in intensity while the fifth is held constant. A typical camera frame capturing the scene is simulated by a frame of *n* × *n* pixels, that has within it, five non-overlapping sub-regions of size *m* × *m* pixels, corresponding to the direct image of the sources in Fig. 1a. A number (N) of such frames are created to represent subsequent snapshots separated by short time intervals (Δt); the intensities in four of the sub-regions are varied sinusoidally as (0.5 + *sin*[*ω*_*i*_*t*_*s*_]), *i* = 1,.. 4 and *t*_*s*_ = *s*Δ*t*, where *s* = 1, 2, … *N*. The arrival of diffusive photons is simulated by adding to every pixel in each frame a (different) random number, uniformly distributed between 0 and X, where X depends on the strength of scattering (simulations were carried out for X ranging from 1 to 10). This results in a series of noisy frames like the one in Fig. 1b, where the sources cannot be directly seen, even though information about the modulated sources is contained in it. From the sequence of *N* such frames, time series are generated for each of the *n*^2^ pixels, by selecting the intensity values for that pixel from successive frames recorded at time instants *t*_*s*_, i.e., an array *I*(*j, k, t*_*s*_), *s* = 1, 2, … *N* was formed for every pixel (j, k) of a frame. The contents of these *n*^2^ time series simulate intensity information of light due to spurious unmodulated ambient illumination, diffusive light from the source, a minute additional sinusoidal contribution simulating the arrival of ballistic photons for pixels in the sub-regions *i*, and also electronic noise. The aim is to extract the ballistic component from the source of interest.

Using a Dell Precision T-3600 desktop computer and programming in MATLAB, we have compared the performance of FFT and QLD techniques of extraction of the ballistic photons. For the former, the FFT function was used, and 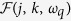, (*q* = −(*N* − 1)/2,.. 0, 1, … *N*/2), the Fourier transform of *I*( *j, k, t*_*p*_) (with p = 1, 2, ……. N) was evaluated. The map of the 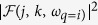 gave the image with the ballistic component that retained modulation at frequency *ω*_*i*_, i.e.,Fig. 1c. In the case of QLD, the quantity 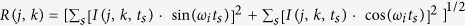 was evaluated for each pixel, (j, k), with the values of sin(*ω*_*i*_*t*_*s*_) and cos(*ω*_*i*_*t*_*s*_) being read from pre-calculated arrays. The 2-d plot of R(j, k) gives the image due to the ballistic photons (Fig. 1d). Both calculations were performed for the same set of recorded frames, and for the same length of time series.

### Estimation of Contrast-to-noise-ratio

**Figure 6:**
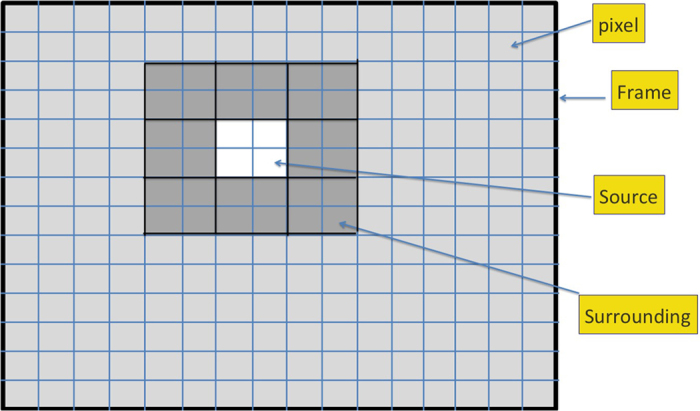
Example of a frame with 14 × 16 pixels, with a source (white region) of size 4 × 4 pixels. The surrounding region considered for determining CNR is made of 8 such 4 × 4 squares, shaded dark grey.

We define^24^ contrast-to-noise ratio as


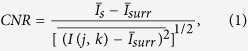


where ‘s’ denotes the source and its intensity *I*_*s*_ is averaged over the *m* × *m* pixels corresponding to the source of interest; ‘surr’ denotes a square slice surrounding the source of interest and its average is perfomed over the 8 *m*^2^ pixels falling in the *m*-pixel wide square slice (see Fig. 6). The sum and average in the denominator is, likewise, performed over these 8 *m*^2^ pixels. The CNR thus provides a measure of how prominent the source is over the surrounding region, in units of the standard deviation of intensity in the surrounding region.

### Estimation of depth of fog

According to ref. 25 moderate fog has a visibility of 125 m; i.e., light flux reduces to 1/e of its initial value over this distance. The scattering medium used had an optical depth of 2.1 and is thus equivalent to 262 m of moderate fog or to 26 m of dense fog. According to current aviation rules regarding Instrument Landing Systems (ILS)^26^ a visibility of 300 m is required for catergory-II landing, and of 175 m for category-IIIA landing. The experiments thus simulate situations where even category-III landing cannot be permitted in case of moderate fog. We show that using QLD and parallel processing, real-time images can be obtained over 262 m of moderate fog, thus increasing visibility. This is illustrated in Fig. 3g, where on performing QLD the runway lights come into view. Once again, QLD at the incorrect frequency yields no image as illustrated in Fig. 3h.

## Additional Information

**How to cite this article**: Sudarsanam, S. *et al*. Real-time imaging through strongly scattering media: seeing through turbid media, instantly. *Sci. Rep.* **6**, 25033; doi: 10.1038/srep25033 (2016).

## Supplementary Material

Supplementary Information
