# Supplementary material for: Real-time imaging through strongly scattering media: seeing through turbid media, instantly
Source: Sci Rep. 2016 Apr 26;6:25033. doi: 10.1038/srep25033 (PMC4844949; doi:10.1038/srep25033)
Supplement: Supplementary Information [file srep25033-s1.pdf]

Real-time imaging through strongly scattering media: seeing through turbid media, instantly : Supplementary Information

Sriram Sudarsanam[1], James Mathew[1], Swapnesh Panigrahi[2],
Julien Fade[2], Mehdi Alouini[2], and Hema Ramachandran[1].
[1]*Raman Research Institute, Sadashiv Nagar, Bangalore, INDIA 560080*
[2]*Institut de Physique de Rennes, Universite de Rennes 1 CNRS, Campus de Beaulieu, 35042 Rennes, FRANCE*

Details of apparatus : We used an ANDOR Neo sCMOS camera that has 2560 x 2160 pixels and is capable of acquiring, in a sustained mode, full frames at 20fps, and smaller frames of 128 x 128 pixels at 1500fps. Data is transferred to the computer over a Camera Link cable. The camera was controlled by our C++ program, using commands provided in ANDOR's software development kit. The camera was maintained at room temperature. Intensity data was recorded with 16-bit resolution.

The computation was carried out on a Dell Precision T3600 desktop computer that has a Xeon Quad Core processor running at 3.4 GHz, equipped with 64GB RAM. Hyperthreading has been enabled in the four CPUs, so that a total of 8 threads can run simultaneously. However, at a time only six were used, as the computer was found to overheat beyond that. The GPU of the desktop was QuadroPro 2000 running at 800 MHz and had 96 CUDA cores. No specialised hardware was used. In fact, the same GPU was also used by the operating system for display. Likewise, all parallelisation functions used were from the OpenSource library, e.g., Open-Multicore-Processing (OpenMP) for data parallelization in CPU, Portable Operating System Interface - POSIX (pThreads) library for task parallelization in CPU and CUDA for utilizing the computational power of the GPU. All computations were performed in double precision.

Comparison of computational times for QLD and FFT : Using a single CPU and using MATLAB (without parallelisation), a comparison was made of the computational times for performing the QLD and FFT operations for different frame sizes and lengths of time series. The results are shown in Fig. 1. QLD is found to have a slight advantage over FFT, for the same input data. This slight advantage, however, acquires significance when we recognise the fact that to produce images with a given contrast-to-noise ratio, shorter time series suffice for QLD when compared to FFT.

When using C++ instead of MATLAB for performing QLD, considerable speedup was observed. Processing times dropped down from 340s to 45s for 800 full-frames (Fig. 2).

Timing sequence for hybrid parallelisation : While the camera has its own software for setting the parameters and acquiring data, considerable time benefit was achieved by writing our own C++ codes. To obtain real-time images, raw-data frames must be processed as they arrive to the system from the camera. To facilitate this, the stream of data that the camera sends must also be stitched and arranged, facilitating processing. The following is the sequence adopted. (See Fig. 3)

- The program begins with the main thread (T1) declaring the necessary variables and setting the physical parameters specified by the user. These include the temperature of the camera, exposure time, and the sampling frequency. The thread then allocates the memory space in the Random Access Memory (RAM) in accordance

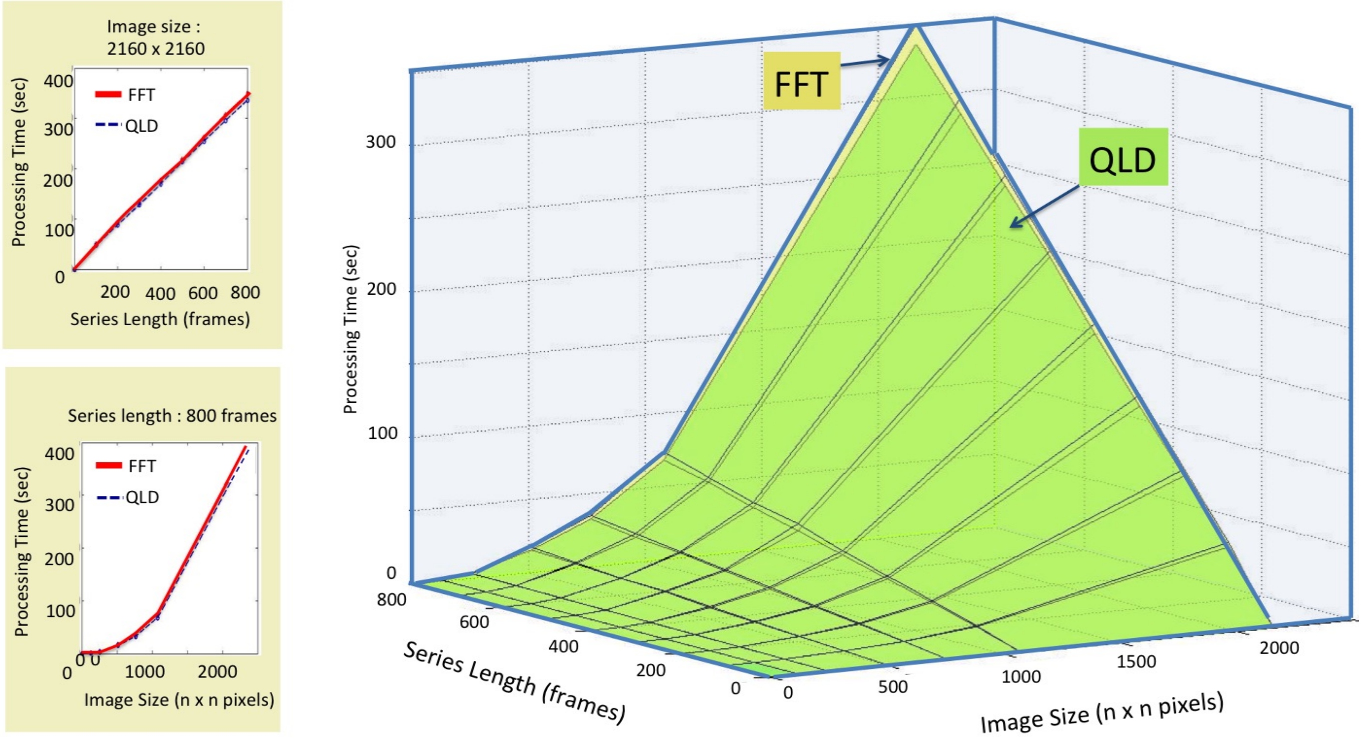

FIG. 1: *Comparison of the processing times for QLD and FFT, using MATLAB for computation.*

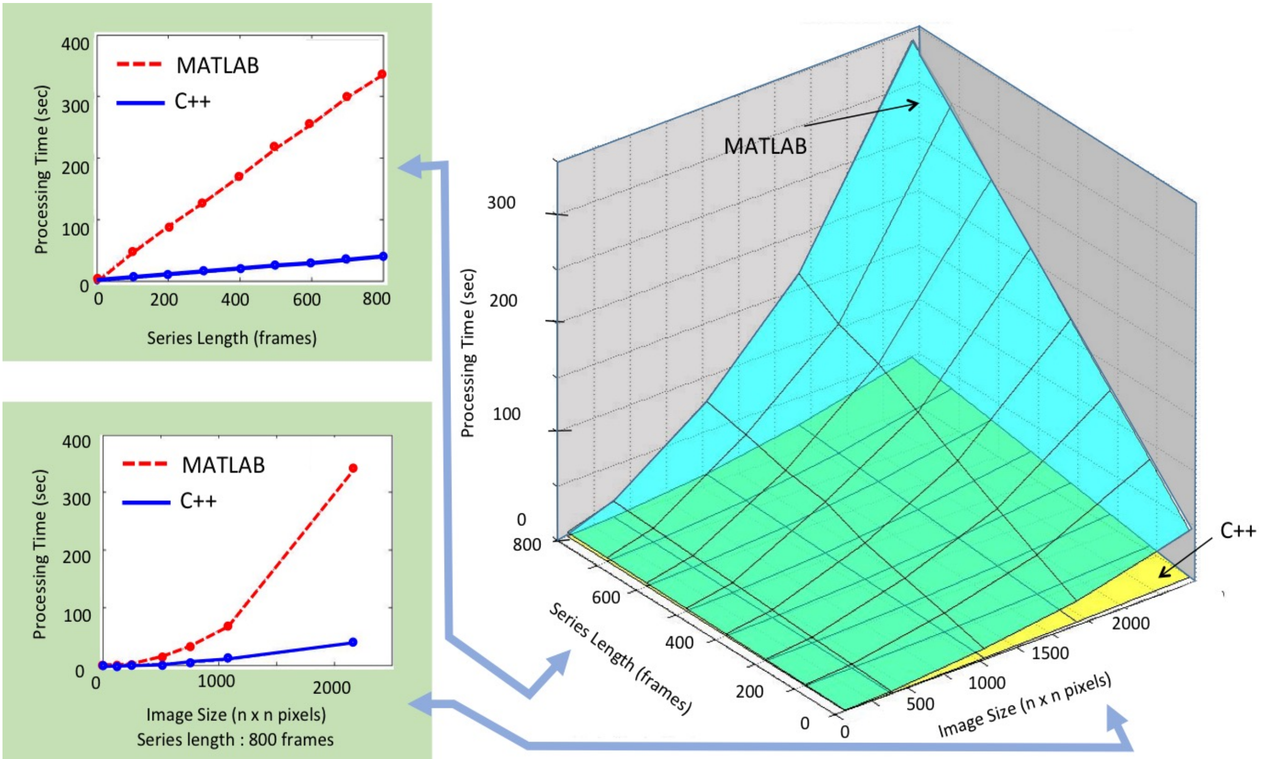

FIG. 2: *Comparison of the processing times for QLD using MATLAB and C++ for computation.*

with the parameters chosen and creates tables of sine and cosine functions, appropriate for the choice of lock-in

and sampling frequencies. Thereafter acquisition is initiated. A stream of 16-bit binary data (2-bytes) now emerges from the camera and is carried over the Camera-link cable to the computer.

- As bytes of data stream in, thread T2 accesses the buffer to stitch pairs of bytes and store values in an array of the appropriate data type to form the raw frame matrices. Upon completion of the task, a flag is set to indicate that the data is in a form ready for analysis.
- Thread T3 invokes the Complete Unified Device Architecture (CUDA) kernel, which prompts the compiler to execute a certain block of program in the cores of the GPU. As the GPU does not have direct access to RAM, thread T3 fetches data (raw frames); this transfer takes place over the PCI express bus at about 100Mbps. Intensity data at each pixel is multiplied by sin and cos to obtain two intermediate frames. Successive intermediate frames are accumulated into two different 2-D arrays that represent the two quadrature components. After accumulation of the required number of terms, the components are squared and added, resulting in the 32-bit $n \times n$ processed image. This is then displayed on the monitor using Open Source Computer Vision (OpenCV).
- Processed data may be stored to the harddisk, if required.
- Flags, indicative of the current status of the threads, are set to ensure proper communication between the threads and to avoid race conditions.
- Although it appears that only three threads are running, each of the threads spawn further threads to facilitate data parallelization. For example, four hardware threads arrange the bytes and arrange the raw data, while the fifth communicates with the graphics card. The sixth hardware thread runs the main sequence.

With multi-threads in the CPU simultaneously performing other (non-computational) tasks, it is now possible to acquire and to process the images simultaneously. This powerful technique allows one to obtain results in milliseconds, faster than the persistence of images in the human eye. The timing sequence is shown in Fig. 3 for the set of parameters used in the table-top experiments described in the main text. Here frames of size 600 x 600 pixels are acquired at 100fps and QLD is performed on data collected over 50ms. The first processed image appears in less than 55ms from the start of acquisition of the first frame and within 5ms of acquisition of the last frame. Processed images appear at the same rate as acquistion. Clearly, using data parallelization in GPU, we can process N frames to obtain one single image *much before* the next frame (i.e., the $(N+1)^{th}$ frame) is acquired. One would expect the processing would fail to complete when the inter-frame interval is small. However, the limitation arises due to the camera, rather than the processing - for any given frame-size, the camera has a limit on the frame transfer rate; the resulting inter-frame interval is much longer than the time needed for processing. Using a sliding sequence, we display frames of size 600×600 pixels at 100fps, thus providing a continuous, flicker-free display of processed images. This rate reduces as the number of pixels per frame increases; for the full 2560×2160 pixels, a processing rate of 20fps is achieved.

In addition to the numerous advantages of QLD mentioned in the main text, we point out here several features that makes QLD particularly suited for GPU-parallelisation. The CUDA FFT library has functions that can only be called from host, and thus the program is limited to the number of CPU threads, in this case 8. Writing our own kernel for QLD allowed us to execute all of the multiplication and accumulation for every pixel at one go. In addition, this

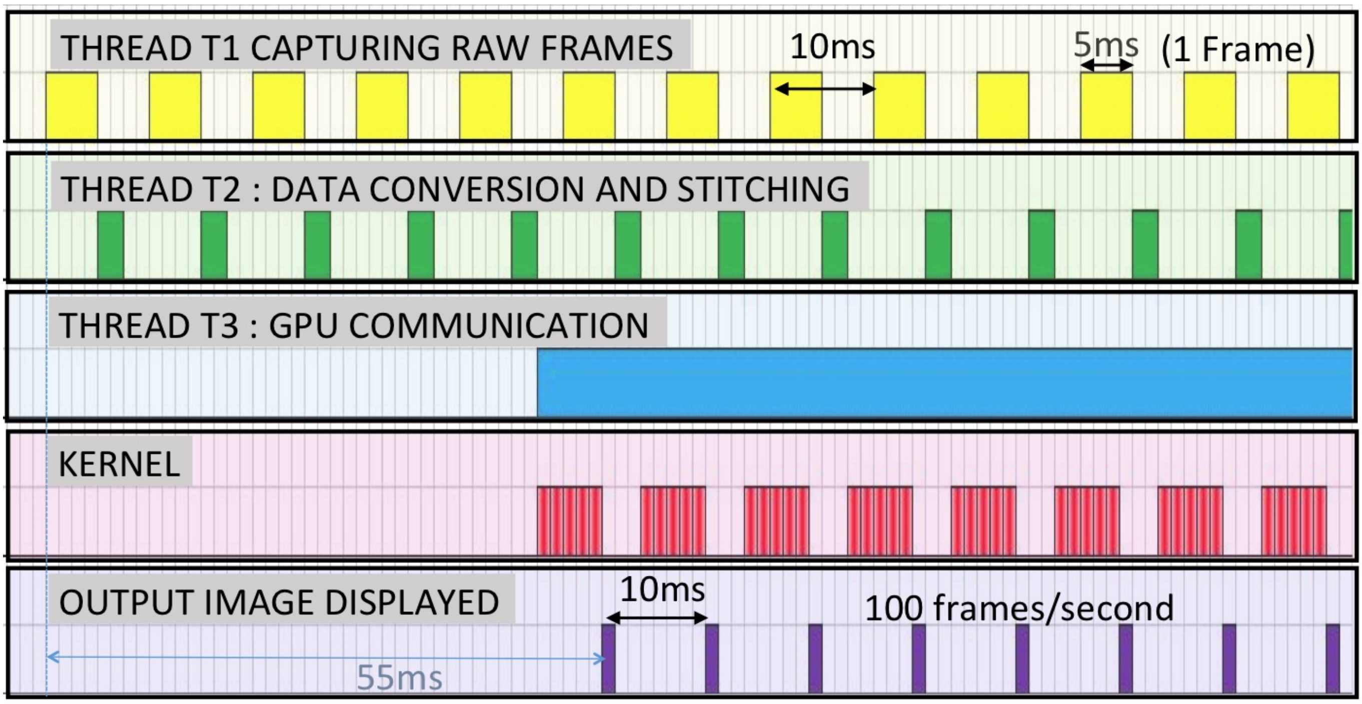

FIG. 3: *Timing sequence for various constituents of the parallel program. The times were obtained from an actual run of the experiment using the equipment described in the text. In this example frames were acquired over 5ms duration, at 100fps. The processed images were displayed at the same rate.*

approach allowed us to free other CPU threads for their respective activities rather than to them await the result, as is the case for CUDA FFT. Another distinct advantage of QLD is that it requires a single buffer that can store one frame (n^2 values), while FFT, on the other hand, requires three buffers: an input buffer, a process buffer and an output buffer, which scale in size with the length of the time series. If a sliding window of length N is used, the buffer requirement for QLD is increased to Nn^2, but this is still a factor of 3 smaller than what would be required for FFT.
